# The effects of switching from 10 to 5-dose vials of MR vaccine on vaccination coverage and wastage: A mixed-method study in Zambia

**DOI:** 10.1016/j.vaccine.2020.07.012

**Published:** 2020-08-18

**Authors:** Kirstin Krudwig, Barbara Knittel, Ali Karim, Natasha Kanagat, Wendy Prosser, Guissimon Phiri, Frances Mwansa, Robert Steinglass

**Affiliations:** a John Snow, Inc, United States; b Ministry of Health, Zambia, Immunization Program, Zambia

**Keywords:** Measles, Doses per container, Multi-dose vial, Immunization coverage, Household survey, Wastage, Health worker

## Abstract

**Introduction:**

Vaccines procured for low-income countries are often packaged in multi-dose vials to reduce program costs. To avoid wastage, health workers may refrain from opening a vial if few children attend an immunization session, possibly leading to lower coverage. Lowering the number of doses in a vial may increase coverage and reduce wastage.

**Methods:**

We used a mixed methods approach to measure the effects of switching from conventional 10-dose measles containing vaccine (MCV) vials to 5-dose MCV vials on coverage and open vial wastage in 14 districts purposely selected from two provinces in Zambia. The districts were paired based on the number of health facilities and the average size of the health facility catchment population. One district from each pair was randomly allocated to receive 5-dose vials while the other continued with the conventional vials. We applied propensity score matched difference-in-difference analysis to estimate intervention effects on coverage using pre-intervention household survey and post-intervention household survey after 11 months of the intervention. The intervention effects on wastage rates were estimated from multivariate analysis of the administrative data. Key informant interviews were conducted to better understand health workers’ behavior and preferences at baseline, midline and endline, and analyzed using thematic analysis techniques.

**Results:**

MCV coverage rates increased across both arms for both doses. A five percentage-point intervention effect was detected for MCV1 and 3.5 percentage-point effect for MCV2. The MCV wastage rate was 47% lower in facilities using 5-dose vials (16.2%) versus 10-dose vials (30.5%). Healthcare workers reported being more willing to open a 5-dose vial than a 10-dose vial for one child, as they were less concerned about wastage.

**Discussion:**

Switching 10-dose MCV vials to 5-dose vials improved coverage, decreased wastage, and improved willingness to open a vial. These findings can contribute to strategies for reducing missed opportunities for vaccination.

## Highlights

• Fear of vaccine wastage may lead to lower immunization coverage.
• Mixed method study compared the use of 5-dose and 10-dose vials of measles-rubella.
• Districts using 5-dose MR saw increase coverage and reduced wastage.
• Health workers reported more willing to open a 5-dose MR vial.
• Switching to 5-dose MR vials can be accommodated within existing cold chain capacity and the wastage-adjusted cost differential per dose is negligible.

## 1 Introduction

Low- and middle- income (LMIC) countries have traditionally procured vaccines in multi-dose vials in order to reduce costs, cold chain storage and distribution requirements [1]. However, higher wastage may offset these benefits as multi-dose vials of vaccines without preservatives (i.e., BCG, measles-containing vaccine [MCV] and yellow fever) must be discarded six hours after reconstitution or at the end of an immunization session, whichever comes sooner [2]. Frontline healthcare workers (HCWs) must decide when to open a vial knowing that doses may be wasted if not enough eligible children are currently present or likely to be present during the course of the session. MCV is usually procured in 10 dose vials for nationwide immunization programs in LMICs. Measles outbreak investigation reports have identified that HCW fear of high MCV wastage was a major contributing factor to low coverage and disease outbreak [3], [4], [5]. In 2018, global coverage with the first dose of MCV was estimated at 86%, the second dose estimated at 54% [6]. This falls short of the goal established by the Global Measles and Rubella Strategic Plan of ≥95% coverage with two doses of measles vaccine in all communities and countries in order to achieve measles elimination [7].

Reducing the number of doses in the vaccine vial is one potential strategy to achieve both high coverage and low wastage in routine immunization programs. Vials with fewer doses may encourage HCWs to vaccinate every eligible child present on both scheduled and unscheduled vaccination days without fearing excess wastage. The major vaccine procurement agency of LMIC programs, UNICEF, made MCV in 5-dose vials available for procurement by country programs in 2018 [8]. Although modeling has shown that vials with fewer doses may be a feasible solution to increasing coverage without placing burdens on the cold chain or cost of an immunization program [9], empirical evidence is lacking [10]. To address the gap, this study was conducted using quantitative and qualitative methods. It was part of the larger Dose Per Container Partnership (DPCP), designed to assess the trade-offs between cost and immunization systems impact choosing vial size.

## 2 Methods and materials

### 2.1 Study context

The Zambian Expanded Programme on Immunization (EPI) has been vaccinating children against measles since the late 1970s. In 2013, a second dose of measles vaccine was introduced to the routine immunization schedule. Measles-containing vaccine (MCV) is now scheduled to be given at nine and 18 months of age. In June 2017, Zambia’s EPI switched from 10-dose monovalent measles to 10-dose measles and rubella (MR) vaccine for both doses in the routine immunization program. Over the past decade, reported measles first-dose vaccination coverage (MCV1) by 12 months of age has fluctuated from 89% in 2008 to 80% in 2013 and to 96% in 2017 [11]. While reported coverage for MCV1 was 96% in 2017, reported second-dose coverage (MCV2) was only 64%. There were also considerable disparities between regions and districts across Zambia, with reported MCV1 district coverage ranging from 64% to 256% in 2017. The 2013–14 Zambia Demographic and Health Survey (DHS) estimated MCV1 coverage for surveyed children 12–23 months of age at 70% (through review of vaccination cards only) and 85% (through review of cards plus a card-adjusted recall of caregivers). Only 73% of the surveyed population had been vaccinated by 12 months of age (card plus recall).

MCV and other vaccines in the routine immunization system are provided at fixed and outreach sessions. Zambia has been procuring 10-dose MCV vials from UNICEF; the long-time availability of the pre-qualified 5-dose MCV on the global market, newly available from UNICEF, provides the context for the study to inform policy.

### 2.2 Study domain

**Fig. 1:**
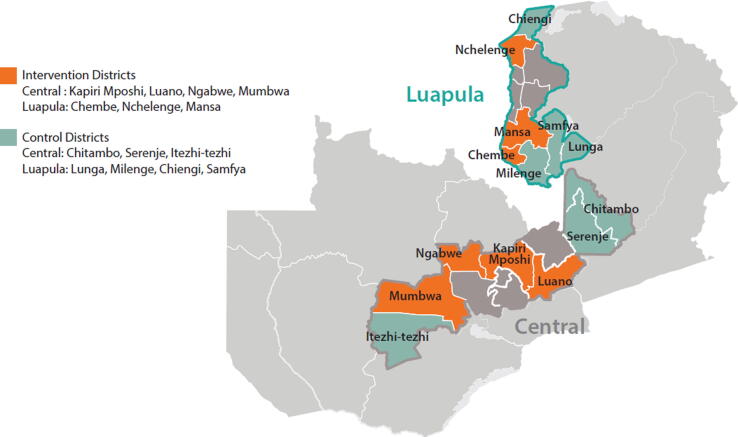
Map of Central and Luapula provinces, Zambia.

The Zambia Ministry of Health in discussions with the study team selected Luapula and Central provinces (Fig. 1) for this study based on comparatively low coverage for Diphtheria-Tetanus-Pertussis 3 (DTP3), MCV coverage, fully immunized child coverage (FIC), timeliness of MCV vaccination among infants; and inequity in wealth, antenatal visits, and maternal education as reported in the 2013–14 DHS. The number of districts in the study was restricted to 14 of 22 districts in the two provinces based on the availability of 5-dose vials of MR vaccine supplies for 12 months. The 14 districts were selected based on number of healthy facilities per district; average target population per health facility (mix of smaller and larger target populations serviced by facility); mix of urban and rural areas; and accessible and reliable data currently collected at district level (see Supplemental Material A for details).

### 2.3 Study design

A mixed-methods design using qualitative and quantitative methods was used to assess the impact of the intervention implemented from June 2017 to April 2018. An intervention-control group design with pre- and post-intervention household surveys was used to measure the effect of the intervention on measles coverage using difference-in-difference analysis. Administrative data were collected from health facilities (HF) within the study area during the intervention period. Indicators of interest were compared between intervention and control areas to measure the intervention’s effect. The qualitative component was derived from key informant interviews.

A stratified-pair, cluster randomized design was implemented in the selected districts for the household survey and administrative data. The districts were paired according to average population size per HF and the number of HFs within each district. From each pair, the intervention district was randomly selected; the other district in the pair served as the control. All HFs in the intervention districts received 5-dose vials of MR while HFs in the control districts continued using the standard 10-dose vials. All 10-dose MR vials were replaced with 5-dose MR vials at the start of the study in the intervention districts. No other interventions such as policy changes or health education approaches were introduced in either arm.

### 2.4 Study participants

The household survey study participants were caregivers of children within two cohorts—children aged 12–23 months and children aged 24–35 months. For the administrative data, the HFs were the unit of analysis. For the in-depth interviews, study participants were facility-based HCWs, district pharmacists (responsible for vaccine storage and distribution), and district maternal and child health (MCH) coordinators (who supervised facility-based HCWs).

### 2.5 Outcomes of interest

The primary outcomes of interest were the effects of switching from 10-dose to 5-dose MR vials on first and second dose MCV coverage, as measured through a pre and post-intervention household survey, and open vial wastage, as measured through a review of administrative data. Secondary outcomes included the effects of this switch on dropouts, session size and frequency, storage and distribution capacity, and HCW preferences regarding vial presentation.

For coverage and dropout indicators, vaccination information was obtained via two sources: card review and caregiver’s recall. If a vaccination card was not available at the time of the interview or vaccination information was absent, the caregiver was then asked to recall whether the child received a given vaccination.

### 2.6 Data

#### 2.6.1 Household survey

A two-stage cluster survey design was used for the household survey. At first stage, enumeration areas (EAs) from the 2010 census were selected with probability proportional to population size within each of the two study arms. At second stage, four households per cohort (i.e., 12–23 and 24–35 months of age) were selected randomly within each selected EA using the following steps: 1) each EA was divided into four approximately equal segments based on household distribution; 2) beginning from the middle of each segment (and working outward), one household per cohort was randomly selected. EAs selected at baseline were revisited during end-line. The 2015 version of the WHO Vaccination Coverage Cluster Survey Questionnaire was adapted for use in this study [12].

#### 2.6.2 Prospective administrative data review

Pre-intervention administrative data were not analyzed for the study due to incomplete records. Paper tools were designed and implemented to record administrative data during the intervention period from all HFs in the study area to measure HF level indicators not captured in the regular Ministry of Health tools (Table 1). HCWs recorded service data daily if a fixed or outreach session was held. At the end of each month, HCWs also documented MR stock information. The data were submitted monthly to the district. The MCH Coordinator entered the data into an Excel-based template and emailed to the study team. Data were reviewed and cleaned in country on a rolling basis with follow up to districts when forms were incomplete or inconsistent.

**Table 1:** Indicator definitions and data sources.

**Indicator**	**Definition**	**Data Source**
MCV 1 vaccination coverage	% of children 12–23 months and 24–35 months who have received at least one dose of MCV	Household survey
MCV 2 vaccination coverage	% of children 24–35 months who have received more than one dose of MCV	Household survey
Penta1 to MCV 1 dropout	% of children 12–23 months and 24–35 months who received Penta1 and did not receive MCV1	Household survey
MCV1 to MCV2 dropout	% of children 24–35 months who received one dose of MCV but did not receive a second dose	Household survey
Session size	# of children who are vaccinated with MCV1 or MCV2 per session	Administrative data
Session Frequency	# of days per month that any doses of MCV were administered	Administrative data
Cold chain storage capacity	% of HFs that have adequate cold chain storage capacity for vaccine schedule	Administrative data
Open vial wastage rate	% of doses in open vials that are unused and discarded	Administrative data

#### 2.6.3 Key informant interviews

Key informant interviews were conducted at baseline, midline and endline HCWs providing immunizations at facilities, district MCH coordinators, and district pharmacists.

At baseline, 60 interviews were conducted across 14 districts: 32 conducted at five urban and 27 rural health facilities (one per facility), and 28 conducted at 14 district offices (two per office). At midline, 24 interviews were conducted in the seven intervention districts: four in urban facilities and 12 rural facilities (one per facility), and 8 conducted in seven district offices. At endline, 56 interviews were conducted in the intervention districts: 5 in urban facilities and 37 in rural facilities (one per facility), and 14 conducted in seven district offices (two in each district office).

Respondents were selected based on job title and responsibilities in order to understand immunization service delivery. Health facilities were selected to ensure representation across large, medium-sized, and small facilities in urban and rural locations within each district. All interviews were transcribed in Microsoft Word and uploaded to NVivo 11 software. Broad themes of interest were identified during the design of the research protocol and key informant interview tools. Transcripts were analyzed in NVivo 11; relevant sections of text were coded and assigned themes during analysis.

#### 2.6.4 Sample size (household survey)

The sample size for the household survey was estimated to detect a 7-percentage-point higher increase in MCV1 and MCV2 rates between baseline survey and endline survey in the intervention group, as compared to the control group (i.e., difference-in-difference), with 80% power, one-sided alpha error set at 0.05, and cluster survey design effect set at 1.5 using the intraclass correlation coefficient from Zambia 2013–14 DHS [13]. The assumption was that the coverage increased from 50% to 57% in the intervention area but remained unchanged at 50% in the control area. The sample size estimation was done using the methods and tools described by McConnell and Vera-Hernandez [14]. Based on this, the sample size was estimated to be 1952 children from 488 clusters per each age cohort and each study arm during each survey period.

### 2.7 Statistical methods

Stata version 14 was used for all the analyses presented [15]. For each cohort, the distribution of the household sample according to selected background characteristics was compared between the two study arms at baseline and at endline using Wald’s statistics adjusted for cluster survey design. The background characteristics considered were child’s sex, province of residence, urban or rural residence, education, occupation, and age of caregiver, time to travel to the nearest health facility or vaccination site, and wealth quintile. Availability of the immunization card in the household on day of survey was also analyzed.

The wealth quintile was created as a single construct using baseline and endline data. A wealth index score was constructed for each household using principal component analysis of household assets and characteristics such as household items, floor material, main water source, and main toilet type [16]. The combined baseline and endline data were divided into five equal groups based on these scores.

To estimate the adjusted intervention effect, the study arms were balanced by matching intervention area EAs with control area EAs using baseline attributes. The propensity scores were first estimated for each EA using a logit model predicting the probability of an EA to be in the intervention area at baseline. The covariates of the logit model were province, urban or rural, EA-level baseline averages of household wealth, sex, age, education and occupation of the caregiver, and outcome variables of interest. Intervention and control EAs with similar propensity scores at baseline were coded so that they could be identified as similar. To assess the adequacy of the matching, t-tests were performed to ensure that the covariates of the final logit model were not statistically significantly different (p > 0.1) between the intervention and the control EAs, after accounting for the matched EAs.

Finally, intervention effects were estimated from logit models predicting the outcome of interest with indicator variables for study arm, survey period, the interaction between study arm and survey period, and for the EAs that matched between the intervention and control areas (dummy variables) as the predictors. The models were adjusted for survey design using Stata’s survey estimators. Stata’s post estimation ‘margins’ command was used to obtain adjusted estimates of the outcomes of interest according to study arm and survey period and the intervention effects (i.e. difference-in-difference) with 95% confidence interval.

#### 2.7.1 Administrative data

Monthly administrative data were collected from 105 facilities in the control area and 135 facilities in the intervention area. Four facilities did not report during the intervention period and were therefore excluded from analysis. The average monthly reporting rates in intervention and control facilities were 91% and 80%, respectively. The distribution of data by session type (outreach/fixed), distance of HF from district capital, province, location of facility, reporting rate, and facility size were compared between the two study arms using Wald’s statistics adjusted for repeated observations within a facility. The mean of the facility-level average monthly open vial wastage rate over the observation period (i.e., 11 months) was then compared between the two study arms, stratified by session type and facility size. The analyses were adjusted for the time series nature of the data (i.e., health facility level monthly measures over the observation period), province, distance of the health facility from the district, catchment population size of the health facility, and reporting rates. To account for possible dependencies between one month’s report with the next or with the previous, an autoregressive conditional heteroskedasticity (ARCH) model was used with 3 month moving averages of monthly reports from each health facility. Predicted wastage rates by study arm and the differences between the two (i.e., the intervention effect) with 95% confidence interval were calculated using Stata’s post estimation ‘margins’ command. Similarly, the average frequency of MR sessions per month and average number of children vaccinated with MR per session were analyzed.

## 3 Results

### 3.1 Household survey

In both cohorts, background characteristics for the two arms showed notable differences at both survey periods. In particular, there was a statistically significant difference among 12–23 month olds in wealth, location of residence, and time to travel to the closest HF (Table 2). A similar difference was seen among the 24–35 month old cohort (Table 3).

**Table 2:** Background characteristics of sampled 12–23 month olds by study arm and survey period.

	Baseline		p-value	Endline		
	Intervention (N = 1907)	Control (N = 1960)		Intervention (N = 1962)	Control (N = 1965)	p-value
**Background characteristic**	**%**	**%**		**%**	**%**	
**Sex of child**						
Male	49.3%	49.4%	0.953	49.2%	49.5%	0.885
Female	50.7%	50.6%		50.8%	50.5%	
**Residence**						
Rural	79.7%	91.0%	<0.001	79.9%	90.5%	<0.001
Urban	20.4%	9.0%		20.1%	9.5%	
**Wealth**						
Lowest	18.4%	24.4%	<0.001	12.4%	27.9%	<0.001
Second	18.4%	24.5%		17.6%	20.8%	
Middle	19.7%	21.9%		17.1%	19.1%	
Fourth	20.3%	14.3%		25.1%	19.9%	
Highest	23.3%	14.9%		27.8%	12.4%	
**Caregiver’s Education**						
No education	8.7%	13.7%	<0.001	10.8%	19.2%	<0.001
Some primary	44.5%	49.6%		40.8%	48.5%	
Completed primary	15.2%	13.2%		14.8%	9.9%	
Some secondary	22.6%	19.2%		24.4%	17.8%	
Completed secondary	7.2%	3.3%		6.7%	3.7%	
More than secondary	1.8%	1.0%		2.6%	0.9%	
**Caregiver’s Occupation**						
Private/ public sector	2.7%	1.4%	<0.001	3.7%	1.3%	<0.001
Agriculture	52.4%	58.9%		42.8%	48.8%	
Self-employed/ own business	11.5%	8.2%		10.5%	4.7%	
Casual work/ petty trade	5.4%	5.9%		10.6%	12.7%	
Unemployed	28.0%	25.0%		32.3%	32.5%	
**Time to travel to closest health facility or vaccination site**						
<10 min	15.4%	16.1%	<0.001	14.2%	26.7%	<0.001
10 to <30 min	25.8%	29.4%		29.9%	29.1%	
30 to <60 min	21.6%	29.8%		31.6%	29.5%	
1–2 h	31.5%	20.5%		20.2%	12.8%	
>2 h	5.7%	4.2%		4.2%	1.9%	

p-values are from Wald’s statistics testing the difference between intervention and control groups.

**Table 3:** Background characteristics of sampled 24–35 month olds by study arm and survey period.

	Baseline		p-value	Endline		
	Intervention (N = 1920)	Control (N = 1867)		Intervention (N = 1931)	Control (N = 1937)	p-value
Background characteristic	%	%		%	%	
**Sex of child**						
Male	48.2%	47.5%	0.634	45.9%	49.6%	0.022
Female	51.8%	52.5%		54.1%	50.4%	
**Residence**						
Rural	79.6%	91.1%	<0.001	79.5%	90.9%	<0.001
Urban	20.4%	8.9%		20.5%	9.1%	
**Wealth**						
Lowest	15.1%	24.8%	<0.001	11.1%	26.1%	<0.001
Second	19.2%	22.6%		16.3%	20.9%	
Middle	20.2%	22.8%		19.8%	19.3%	
Fourth	20.0%	14.5%		26.0%	19.9%	
Highest	25.6%	15.4%		26.9%	13.8%	
**Caregiver’s education**						
No education	9.3%	14.0%	<0.001	11.0%	18.9%	<0.001
Some primary	46.9%	49.1%		43.7%	46.4%	
Completed primary	16.6%	14.5%		14.8%	12.2%	
Some secondary	20.6%	16.9%		22.6%	17.9%	
Completed secondary	8.2%	4.3%		5.7%	3.1%	
More than secondary	1.4%	1.0%		2.2%	1.6%	
**Caregiver’s Occupation**						
Private/ public sector	3.2%	2.0%	<0.001	3.2%	1.6%	<0.001
Agriculture	53.3%	58.9%		46.0%	49.5%	
Self-employed/ own business	13.7%	10.0%		9.6%	6.5%	
Casual work/ petty trade	5.3%	7.7%		9.7%	13.0%	
Unemployed	24.5%	21.4%		31.5%	29.6%	
**Time to travel to closest health facility or vaccination site**						
<10 min	15.2%	16.0%	<0.001	14.5%	27.5%	<0.001
<30 min	27.0%	30.7%		29.5%	28.8%	
<1 h	21.1%	29.0%		29.5%	29.7%	
1–2 h	30.8%	20.5%		21.5%	12.2%	
>2 h	6.0%	3.9%		5.0%	1.8%	

p-values are from Wald’s statistics testing the difference between intervention and control groups.

Card availability on the day of the interview increased in both cohorts at endline (Table 4). Among children 12–23 months, there was a statistically significant increase in card availability, from about 73% at baseline to 85% at endline in both study arms (p < .001). Children 24–35 months had similar increases in card availability, increasing statistically significantly from 63% at baseline to about 76% at endline for children in both study arms (p < .001).

**Table 4:** Card availability by age of child.

	Baseline			Endline		
	Intervention	Control	p-value	Intervention	Control	p-value
**Card availability for children aged 12–23 months**						
N	1907	1960		1962	1965	
Card available today	73.2%	71.5%	0.32	84.6%	84.8%	0.86
**Card availability for children aged 24–35 months**						
N	1920	1867		1931	1937	
Card available today	63.4%	63.3%	0.97	76.4%	75.3%	0.49

#### 3.1.1 Coverage rates

Balance of the covariates between intervention and control areas at baseline due to matching is given in Table 11 of Supplemental Material B. Adjusted coverage rates for MCV1 based on vaccination cards increased statistically significantly from baseline to endline in both study arms (Table 5). In the intervention arm, MCV1 adjusted coverage rates significantly increased by 14 percentage points from baseline (62%) to endline (76%) (p < .001). Similarly, in the control arm, MCV1 coverage increased from 63% to 77% (p < .001) across the two survey periods. However, the difference in the changes in coverage rates between the two study arms (i.e., the treatment effect or difference-in-difference) were not statistically significant (p = .869).

**Table 5:** Coverage of MCV1 in children 12–23 months and MCV2 in children 24–35 months by data source.

Indicator	Source of data	Study arm	Baseline	Endline	Difference between baseline and endline			Treatment effect			
					diff	95% CI		diff-in-diff	95% CI		p-value
**MCV1 Coverage**	**Vaccination card**	Intervention	61.8%	75.6%	13.8%	11.8%	15.8%	0.2%	−2.3%	2.7%	0.869
		Control	63.1%	76.7%	13.6%	11.8%	15.4%				
	**Vaccination card and caregiver's recall**	Intervention	82.1%	91.6%	9.6%	8.1%	11.0%	4.9%	0.3%	6.6%	<0.001
		Control	84.2%	88.8%	4.7%	3.4%	6.0%				
**MCV2 Coverage**	**Vaccination card**	Intervention	24.3%	39.4%	15.1%	13.1%	17.2%	−0.4%	−3.0%	2.2%	0.777
		Control	25.8%	41.4%	15.5%	13.4%	17.6%				
	**Vaccination card and caregiver's recall**	Intervention	43.0%	55.8%	12.8%	10.7%	14.9%	3.5%	1.0%	6.1%	0.007
		Control	45.0%	64.2%	19.3%	7.2%	11.3%				

MCV1 coverage based on both sources (card review and caregiver’s recall) increased statistically significantly between baseline and endline. A statistically significant intervention effect of about five percentage-points (p < .001) was detected when data from both sources were considered to estimate MCV1 coverage.

Based on card review, there was a statistically significant increase in the adjusted coverage rates of MCV2 among children aged 24–35 months during the survey period across both study arms (Table 5). In the intervention arm, coverage increased from 24% at baseline to 39% at endline (p < .001). Similarly, in the control arm, coverage increased from 26% at baseline to 41% at endline (p < .001). However, the intervention effect was not statistically significant for the adjusted MCV2 coverage from card review (p = .777).

Adjusted coverage of MCV2 based on both sources (card review and caregiver’s recall) increased significantly between pre- and post-intervention in both study arms. A statistically significant intervention effect was detected when data from both sources were considered to estimate MCV2 coverage. A 3.5-percentage-point increase in the adjusted rate for MCV2 coverage in the intervention area is attributable to the intervention (p = .007).

In the intervention arm, there was a statistically significant decrease in adjusted dropout rates for Penta 1 to MCV1, based on children with cards (p < .001) (Table 6). In the control arm, adjusted dropout rates among children with cards also decreased statistically significantly from 12.4% to 6.6% (p < .001). Adjusted dropout rates based on cards and caregiver recall also decreased approximately 7 percentage points in the intervention arm (p < .001) and 4.7 percentage points in the control arm (p < .001). A 2.6 percentage-point reduction in the adjusted dropout rate from Penta 1 to MCV 1 in the intervention area is attributable to the intervention (p = .010).

**Table 6:** Penta 1-MCV1 Drop-out in children 12–23 months and MCV1-MCV2 Drop-out in children 24–35 months by data source.

Indicator	Source of data	Study arm	Baseline	Endline	Difference between baseline and endline			Treatment effect			
					diff	95% CI		diff-in-diff	95% CI		p-value
**Penta 1 – MCV1 Drop-out**	**Vaccination card**	Intervention	13.0%	7.2%	−5.8%	−7.5%	−4.1%	0.01%	−1.9%	2.0%	0.992
		Control	12.4%	6.6%	−5.8%	−7.4%	−4.1%				
	**Vaccination card and Caregiver’s recall**	Intervention	15.3%	7.9%	−7.3%	−9.1%	−5.6%	−2.6%	−4.7%	−0.6%	0.010
		Control	14.0%	9.3%	−4.7%	−6.3%	−3.1%				
**MCV1 – MCV2 Drop-out**	**Vaccination card**	Intervention	44.9%	30.1%	−14.8%	−18.1%	−11.5%	0.3%	−3.7%	4.2%	0.900
		Control	43.3%	28.2%	−15.1%	−18.2%	−12.0%				
	**Vaccination card and Caregiver’s recall**	Intervention	36.4%	21.9%	−14.5%	−17.2%	−11.8%	−3.6%	−6.9%	−0.2%	0.038
		Control	33.8%	22.8%	−11.0%	−13.6%	−8.4%				

There was a statistically significant decrease in the adjusted dropout rates of MCV1 to MCV2 among children 24–35 months pre- and post-intervention in both study arms (Table 6). In both the intervention and control arms, the adjusted dropout rates of MCV1 to MCV2 (based on cards) decreased by approximately 15 percentage points respectively (p < .001 and p < .001). The intervention effect was statistically significant when both cards and caregiver recall was considered (p = .038), attributing to a 3.6-percentage-point reduction in the dropout rate in the intervention area compared to the control area.

### 3.2 Administrative data review

There were no notable differences in HF characteristics between study arms aside from reporting rates (Table 7). Reporting rates were statistically different between the intervention and control arms (p < .001).

**Table 7:** Distribution of administrative data records by health facility characteristics and study arm.

		**Intervention**	**Control**	**P-value**
**Distance to district capital**	0–39 km	49.3%	39.7%	0.302
	40–99 km	40.6%	31.6%	
	>100 km	19.7%	19.1%	
**HF location**	Rural	88.4%	94.4%	0.224
	Urban	5.0%	3.6%	
	Missing classification	6.6%	2.1%	
**HF Size**	Large: 500+ Target Pop	15.6%	13.8%	0.854
	Medium: 200–499 Target Pop	46.2%	44.5%	
	Small: 0–199 Target Pop	38.3%	41.7%	
**Province**	Central	55.1%	46.7%	0.206
	Luapula	44.9%	53.4%	
**Reporting Rate**	<75%	16.1%	3.6%	<0.001
	75–94%	35.8%	29.8%	
	>94%	48.2%	66.6%	
**Total Number of Reports Submitted**		**2708**	**1852**	
**Total Number of Facilities**		**135**	**105**	

There was an intervention effect on the open vial wastage rate, depicted by the significant difference in wastage rates between study arms (30.5% in the intervention arm and 16.2% in the control arm) with the control area HFs having 14 percentage-points higher wastage rates than HFs in the intervention area (p < .001, Table 8). The intervention effect on wastage rates was seen for both fixed and outreach sessions, but the intervention effects did not vary by the session type.

**Table 8:** Wastage rates by study arm, type of vaccination session and health facility size.

				Treatment Effect			
		Intervention	Control	Difference	95% CI		P-value
**Session Type**	Fixed	16.7%	30.5%	−13.77	−17.28	−10.25	<0.001
	Outreach	17.5%	31.2%	−13.68	−16.97	−10.39	<0.001
**Health facility size**	Large: 500+ Target Pop	18.9%	28.2%	−9.32	−16.67	−1.97	0.013
	Medium: 200–499 Target Pop	17.5%	29.4%	−11.85	−16.06	−7.63	<0.001
	Small: 0–199 Target Pop	13.4%	32.4%	−18.92	−23.33	−14.50	<0.001
	Difference between medium vs large effect			−2.53	−11.0	5.95	0.559
	Difference between small vs large effect			−9.59	−18.10	1.07	0.027
	Difference between small vs medium effect			−7.06	−13.14	−0.98	0.023
**Total**		**16.2%**	**30.5%**	**−14.35**	**−17.21**	**−11.49**	**<0.001**

There was a significant variation of the intervention effect on wastage rates by the size of health facility. The treatment effect was seven and 10 percentage-points greater (p < .05) for small health facilities when compared to the treatment effects among the medium and large health facilities.

There was no treatment effect on the average number of sessions per month where MR vaccines were administered (Table 9). This was true for both outreach and fixed sessions. The results indicated that facilities in both study arms were on average conducting statistically significantly (p < .05) more outreach sessions than fixed sessions (analysis not shown in Table 9). The results also show that vial size has little effect on the average number of children immunized with MR vaccine per session. There was no significant difference in number of children vaccinated per session with MR between the control and intervention HFs.

**Table 9:** Average frequency and size of immunization sessions where MR and Penta was given.

	**Intervention**	**Control**	**Difference**	**95% CI**		**P-value**
**Average # of times per month MR vaccines administered**						
Fixed	1.9	1.7	0.2	−0.06	0.44	0.135
Outreach	2.6	2.9	−0.2	−0.59	0.14	0.228
Total	4.5	4.5	0.0	−0.47	0.40	0.876
**Average number of children vaccinated with MR per session**						
Fixed	10.7	10.5	0.3	−1.27	1.80	0.736
Outreach	8.7	10.0	−1.3	−2.40	−0.14	0.027
Total	9.9	10.3	−0.5	−1.60	0.69	0.436

### 3.3 Key informant interviews

Most HCWs stated that their job performance was assessed based on immunization coverage and not vaccine wastage. However, they were concerned about wastage and knew their wastage was monitored. Concerns for wastage and ensuring stock availability until next restocking seemed to influence when to open a vial. When asked whether their practices had changed since the introduction of the 5-dose vial, HCWs replied they were less concerned about MR vaccine wastage and felt more comfortable opening vials to vaccinate children. Thirty-eight of 42 (90%) HCWs using 5-dose MR vials reported offering MR vaccines at every fixed session regardless of the number of eligible children presenting. By contrast, at baseline over 50% of respondents using 10-dose vials indicated that they waited for a minimum of five children before offering the MR vaccine. All 42 HCWs using 5-dose MR vials reported offering MR vaccines at every outreach session. At baseline and endline, on days when sessions were not scheduled, HCWs in both study arms reported asking mothers to return with their child when a session was scheduled. If families came from great distances, HCWs using 5-dose vials reported opening a vial and vaccinating the child regardless of the potential impact on vaccine wastage.

None of the HCWs using the 5-dose MR vials wanted to return to using the 10 dose MR vials at the end of the study. They all reported that using 5-dose vials had positively influenced their ability to vaccinate more children and reduced their concern about wastage.

## 4 Discussion

This study looked at the effects of switching from 10-dose to 5-dose MR vials on coverage, drop-outs, wastage, session size and frequency, and on HCW behavior. The study design of the household survey increased both validity and efficiency of the study sample through revisiting the EAs from baseline during the endline, the analytic method of matching intervention area EAs with control area EAs, or the combination of both [17], [18]. The findings suggest that 5-dose vials of MR can contribute to improving coverage, reduce drop-outs, reducing wastage, and influencing HCW behavior. With HCWs more willing to open a vial with fewer doses, this change may contribute to reducing missed opportunities in vaccination (MOVs) [19].

Despite national guidance to open a vaccine vial for every eligible child, it is clear that decisions around when to open an MR vial often take into account HCW concerns for limiting wastage. In the qualitative research, HCWs stated that they wait for an average of five children to open a 10-dose MCV vial. It is promising that HCWs indicated through this study that they are more willing to open a vial for only one child when using 5-dose vials.

This HCW behavior change had an effect on reducing open vial wastage with 47% lower wastage rate in facilities using 5-dose MR vials versus 10-dose MR vials. Responses from HCWs corroborate these results as they also stated that the reduction in wastage is a major benefit of using 5-dose vials. Providers in the intervention arm overwhelmingly stated their preference to continue using 5-dose MR vials.

Increased coverage attributable to the 5-dose vials is a noteworthy research finding, even as MR coverage rates have been increasing continuously nationwide in Zambia over the past several years. The 4.9% increase in MR1 and 3.5% increase in MR2 suggest that more children are being vaccinated when HCWs have access to 5-dose vials. The post-intervention household survey showed that while coverage rates increased across both arms, an intervention effect was still detectable in the intervention arm in both cohorts when adjusted for confounding factors. The reduced dropout rate implies improved service quality with more children completing the course of vaccines. It is interesting to note that the data showed no difference in frequency of providing MR vaccine between intervention and control facilities. Card coverage also increased across arms due to extraneous factors that cannot be explained by this study.

The fear of open vial wastage of high volume vaccines without preservatives can be a barrier to ensuring all children are vaccinated. Immunization programs can employ multiple strategies to reduce MOVs during routine immunization, such as decision support tools and job aids for HCW, integrating curative and preventive services, or shifting workflow or immunization session frequency. As demonstrated by this study, smaller vial size can be considered another strategy to reduce MOVs as well as reduce wastage. This study was conducted through routine immunization services; supplemental immunization activities such as mass campaigns may have different considerations for optimal vial size.

No policy changes or directions to HCWs on use of the 5-dose vials were introduced in either arm of the study. Based on study results, it is likely that the introduction of the 5-dose MR vial for routine immunization programs, accompanied by reminders to HCWs to open a vial for even a single eligible child, would over time likely increase positive changes on vaccination coverage, drop out, missed opportunities, and vaccine wastage.

The coverage increase that was detectable in a country with high overall coverage suggests that coverage increases in other settings with lower coverage may also be possible by using a vial with fewer doses. UNICEF made 5-dose MCV vials available for procurement by country programs in 2018. The study concludes that the higher wastage-adjusted purchase price per dose from UNICEF for monovalent measles in 5-dose versus 10-dose vials (1.6 US cents) and for MR vaccine in 5-dose versus 10-dose vials (3.2 US cents) is negligible. With the evidence suggesting positive effects on coverage and wastage, it is an opportune moment for countries to consider different vial size presentations.

## 5 Limitations

During the second stage of the household survey, the selection of households and respondents was not based on probability but a quasi-random process which can give biased estimates. Since potential bias due to second stage sampling is similar between the two study arms and between the survey periods, and that the EAs that were sampled in baseline were revisited during the endline, the difference-in-difference analysis cancels this out. In other words, the intervention effect estimates are unlikely to be biased due to the sampling strategy.

The likelihood of imbalance between the intervention and control groups in the analysis of the household survey was minimized by applying propensity scores to match intervention EAs with control EAs at baseline. Nonetheless, imbalance could still be present due to unobserved confounders.

Masking of the intervention area health facilities was not possible. However, there is no reason to believe that intervention health facilities received comparatively better monitoring and supervision, as there were no additional resources provided to do so. It is unlikely that the district administration of the intervention districts systematically provided greater resources on supervision compared to those of the control districts. The study team, however, had frequent remote communication with the health facilities and district supervisors of both study arms to obtain administrative data, which may have facilitated the managers and service providers to better use data to improve performance.

Due to lack of usable pre-intervention data, it is possible that the intervention effects observed from the administrative data are partly due to unknown pre-intervention differences between the two study arms.

The intervention effects estimated from the household survey were based on intention to treat analysis. Thus, the treatment effects could be underestimated due to contamination (if some of the children in the intervention districts attended immunization sessions in the adjacent district where 10-dose MR vials were used, or vice versa).

## 6 Ethics approval

This research protocol was developed in collaboration with the Zambian Ministry of Health, Dose Per Container Partnership (DPCP) partners, DPCP Technical Advisory Group, and the Bill & Melinda Gates Foundation (which funded the research). The study was approved by the Biomedical Research Ethics Committee of the University of Zambia, as well as by the Institutional Review Board (IRB) of JSI Research & Training Institute, Inc.

## Funding

This work was supported by the 10.13039/100000865Bill & Melinda Gates Foundation, Seattle, WA [OPP1140568]. The 5-dose MR used in the study was donated by the Serum Institute of India.

## CRediT authorship contribution statement

**Kirstin Krudwig:** Methodology, Data curation, Formal analysis, Writing - original draft, Writing - review & editing. **Barbara Knittel:** Data curation, Formal analysis, Writing - original draft, Writing - review & editing. **Ali Karim:** Methodology, Formal analysis, Validation. **Natasha Kanagat:** Methodology, Data curation, Formal analysis. **Wendy Prosser:** Writing - review & editing, Visualization. **Guissimon Phiri:** Supervision, Visualization, Writing - review & editing. **Frances Mwansa:** Supervision, Methodology. **Robert Steinglass:** Conceptualization, Methodology, Writing - review & editing, Supervision, Funding acquisition.

## Declaration of Competing Interest

The authors declare that they have no known competing financial interests or personal relationships that could have appeared to influence the work reported in this paper.
