# Supplementary material for: The effects of switching from 10 to 5-dose vials of MR vaccine on vaccination coverage and wastage: A mixed-method study in Zambia
Source: Vaccine. 2020 Aug 18;38(37):5905–13. doi: 10.1016/j.vaccine.2020.07.012 (PMC7427328; doi:10.1016/j.vaccine.2020.07.012)
Supplement: Supplementary data 1 [file mmc1.doc]

**The effects of switching from 10 to 5-dose vials of MR vaccine on vaccination coverage and wastage: A mixed-method study in Zambia**

**Appendix A**

Table 10. District selection criteria

| **District Name** | **#HF** | **Avg Target pop**  **per HF** | **# of urban HFs** | **# of rural HFs** |
| --- | --- | --- | --- | --- |
|
|  |
| **Central Province** | | | | |
| Chibombo | 30 | 407 | 1 | 29 |
| Chisamba | 11 | 196 | 2 | 9 |
| Chitambo | 12 | 118 | 1 | 11 |
| Itezhi-tezhi | 17 | 213 | 3 | 14 |
| Kabwe | 41 | 223 | 36 | 5 |
| Kapiri-Mposhi | 31 | 394 | 5 | 26 |
| Luano | 11 | 94 | - | 11 |
| Mkushi | 26 | 260 | 2 | 24 |
| Mumbwa | 30 | 273 | 4 | 26 |
| Ngabwe | 3 | 281 | - | 3 |
| Serenje | 21 | 308 | 4 | 17 |
| **Luapula Province** | | | | |
| Chembe | 5 | 309 | - | 5 |
| Chiengi | 12 | 502 | 1 | 11 |
| Chipili | 14 | 129 | - | 14 |
| Kawambwa | 20 | 230 | 2 | 18 |
| Lunga | 4 | 210 | - | 4 |
| Mansa | 33 | 308 | 6 | 27 |
| Milenge | 9 | 273 | - | 9 |
| Mwansabombwe | 7 | 341 | 1 | 6 |
| Mwense | 14 | 287 | 2 | 12 |
| Nchelenge | 17 | 475 | 1 | 16 |
| Samfya | 32 | 286 | 5 | 27 |

**Appendix B**

Table 11. Baseline adjusted and unadjusted estimates of outcome variables

| **Baseline adjusted and unadjusted estimates of the outcome variables of interest** | | | | | | |
| --- | --- | --- | --- | --- | --- | --- |
| **Indicators** | **Unadjusted** | | | **Adjusted** | | |
|  | **Intervention** | **Control** | **p-values** | **Intervention** | **Control** | **p-values** |
| MCV1 coverage (according to vaccination card among children 12-23 months) | 61.7% | 61.6% | 0.961 | 60.7% | 62.9% | 0.273 |
| MCV1 coverage (according to vaccination card and caregiver recall among children 12-23 months) | 83.3% | 83.1% | 0.870 | 82.8% | 83.0% | 0.921 |
| MCV2 coverage (according to vaccination card among children 24-35 months) | 29.2% | 28.2% | 0.528 | 28.9% | 28.7% | 0.935 |
| MCV2 coverage (according to vaccination card and caregiver recall among children 24-35 months) | 51.5% | 58.0% | 0.003 | 54.2% | 53.9% | 0.878 |
| Penta 1 – MCV1 Drop-out (according to vaccination card among children 12-23 months) | 15.4% | 13.2% | 0.120 | 15.4% | 13.4% | 0.232 |
| Penta 1 – MCV1 Drop-out (according to vaccination card and caregiver recall among children 12-23 months) | 16.3% | 16.5% | 0.844 | 16.8% | 16.5% | 0.847 |
| MCV1 – MCV2 Drop-out (according to vaccination card among children 24-35 months) | 47.6% | 49.5% | 0.426 | 47.8% | 49.2% | 0.604 |
| MCV1 – MCV2 Drop-out (according to vaccination card and caregiver recall among children 24-35 months) | 41.0% | 35.3% | 0.003 | 38.6% | 38.9% | 0.895 |
| Province | 56.9% | 40.0% | <0.001 | 53.8% | 51.2% | 0.500 |
| Residence (Urban/Rural) | 79.6% | 91.0% | <0.001 | 77.2% | 74.9% | 0..537 |
| Caregiver’s Occupation | | | | | | |
| Agriculture | 52.9% | 58.9% | 0.003 | 56.3% | 53.7% | 0.205 |
| Unemployed | 26.3% | 23.2% | 0.029 | 24.4% | 26.1% | 0.302 |
| Caregiver’s Education | | | | | | |
| Some Primary | 44.2% | 49.4% | <0.001 | 46.7% | 45.6% | 0.479 |
| Completed Primary | 15.9% | 13.8% | 0.035 | 14.8% | 15.5% | 0.533 |
| Some Secondary | 21.6% | 18.1% | 0.002 | 20.1% | 20.0% | 0.810 |
| Secondary or higher | 9.4% | 4.8% | <0.001 | 7.5% | 8.5% | 0.273 |
